# Identification of factors related to food insecurity and the implications for social determinants of health screenings

**DOI:** 10.1186/s12889-021-11465-6

**Published:** 2021-07-16

**Authors:** Ashley R. Banks, Bethany A. Bell, David Ngendahimana, Milen Embaye, Darcy A. Freedman, Deena J. Chisolm

**Affiliations:** 1 Center for Child Health Equity and Outcomes Research, The Abigail Wexner Research Institute at Nationwide Children’s Hospital, 700 Children’s Drive, Columbus, OH 43205 USA; 2 School of Education and Human Development, University of Virginia, Charlottesville, VA USA; 3 Department of Population and Quantitative Health Sciences, Case Western Reserve University, Cleveland, OH USA; 4 Department of Pediatrics, The Ohio State University College of Medicine, Columbus, OH USA

**Keywords:** Social determinants of health screening, Food insecurity, Health disparities

## Abstract

**Background:**

Food insecurity and other social determinants of health are increasingly being measured at routine health care visits. Understanding the needs and behaviors of individuals or families who screen positive for food insecurity may inform the types of resources they need. The goal of this research was to identify modifiable characteristics related to endorsement of two food insecurity screener questions to better understand the resources necessary to improve outcomes.

**Methods:**

Analysis was conducted focusing on cross-sectional survey data collected in 2015–2016 from participants (*N* = 442) living in urban neighborhoods in Ohio with limited access to grocery stores. Food insecurity was assessed by the endorsement of at least one of two items. These were used to categorize participants into two groups: food insecure(*N* = 252) or food secure (*N* = 190). Using logistic regression, we estimated the association between several variables and the food insecure classification.

**Results:**

Those that used their own car when shopping for food had lower odds of reporting food insecurity, as did those with affirmative attitudes related to the convenience of shopping for and ease of eating healthy foods. As shopping frequency increased, the odds of food insecurity increased. Food insecurity also increased with experience of a significant life event within the past 12 months. There was an 81% increase in the odds of reporting food insecurity among participants who received Supplemental Nutrition Assistance Program benefits compared to those not receiving Supplemental Nutrition Assistance Program benefits.

**Conclusions:**

Along with referrals to SNAP, clinicians can further address screening-identified food insecurity through provision of transportation supports and linkages to other social services while collaborating on community initiatives to promote convenient and easy access to healthy foods. The needs and behaviors associated with screens indicating food insecurity also have implications for impacting other SDH, and thus, health outcomes.

**Supplementary Information:**

The online version contains supplementary material available at 10.1186/s12889-021-11465-6.

## Background

In 2018, approximately 11.1% of all US households and 13.9% of households with children experienced one of the most common social determinants of health (SDH), food insecurity [1]. The US Department of Agriculture (USDA) defines food insecurity in two levels. The first, low food security, represents reduced quality, variety, or desirability of diet yet little or no indication of reduced food intake. The second is very low food security, which includes reports of multiple indications of disrupted eating patterns and reduced food intake [2]. Food insecurity is most often the result of a combination of financial and structural barriers and research demonstrates that it affects the health and well-being of individuals by contributing to higher rates of obesity, fewer heathy foods served at meals, lower quality diets, mental distress, and functional limitations [3–6]. These consequences differ based on demographic characteristics (i.e., race, ethnicity, and age) contributing to health inequities across populations [7, 8]. In addition to these consequences, food insecurity is directly associated with dietary behaviors and perceptions [9, 10]. Despite some food insecure individuals perceiving healthy eating as beneficial to health, many also view it as inconvenient [9]. Balancing a value for healthy eating with the need to stretch food budgets sometimes results in dietary behaviors (i.e., reducing fruit and vegetable purchases) that have negative implications for health [11, 12].

Based on the potential for food insecurity and other SDH to impact health outcomes, screening in primary care settings has been discussed as a means to help providers identify needs and address issues through referrals to additional resources [13]. In 2015, the American Academy of Pediatrics (AAP) recommended screening for food insecurity in children through the use of a two-item screener called the Hunger Vital Sign™ screening tool, which addresses worry about food insecurity and experiencing food insecurity [14]. The Hunger Vital Sign™ has been used in a variety of clinical settings, with mixed results in acceptability by patients and providers and in effectiveness for identification of food insecurity and subsequent referral to resources. Barriers related to using this tool include patient refusals, patient discomfort in talking about food needs with providers, perceptions of ineligibility for supportive services, and challenges in implementation of referral processes for families who indicate a need for food resources [15–17].

Despite these barriers, screening for food insecurity and other SDH in primary care settings may result in more referrals and thus the potential for more individuals or families to access needed community resources [18]. In addition, screening may help providers better understand factors influencing their patients’ health outcomes, and further, identify the need for resources that go beyond provision of food for a family. However, even with the potential value of screening for food insecurity, implementation can have substantial operational cost. The value of such screening is ultimately defined by the usefulness of the data collected. Brief SDH screening items, like those recommended by the AAP and other bodies, provide limited information about a person’s actual needs and little is known about the demographic, attitudinal, or behavioral characteristics associated with a positive screen for food insecurity. The present analysis aimed to identify needs and behaviors related to the endorsement of two food insecurity screener questions like those included in the Hunger Vital Sign™. The goal of this work is not to validate a new screening tool, but instead to identify potential intervention targets in those individuals endorsing screening items.

## Methods

### Study population

This analysis uses baseline data from a longitudinal natural experiment investigating the impact of the food retail environment on dietary behaviors [19]. Participants were recruited from two urban neighborhoods in Cleveland and Columbus, Ohio, each identified as low-income and having low access to healthy food. The two neighborhoods have similar racial and economic composition (i.e., majority African American, approximately 40% received Supplemental Nutrition Assistance Program [SNAP] benefits) and access to healthy food retailers.

Participants were recruited from August 2015 to July 2016 using mailings, flyers, public presentations, and word-of-mouth. Interested individuals were invited to complete the baseline surveys if they were 1) at least 18 years old, 2) living in a targeted census tract, 3) intending to remain in their current neighborhood for at least 12 months, 4) the primary food shopper of the household, and 5) English speaking. A total of 1395 individuals were screened for eligibility, of which 655 were eligible. Ineligibility was mainly due to living outside of the targeted geographic area. Of the eligible participants, 516 completed the three surveys. From the 516, we limited the sample to those who had complete data on the variables to be included in the logistic regression, which are found in Table 2 below, bringing the final sample to 442 participants. Name Redacted Institutional Review Board issued approval for study activities, and participants completed the informed consent process prior to participation.

### Data collection

Data collection occurred from August 2015 to September 2016. Survey data included three 24-h dietary recalls and a psychosocial survey. Surveys and recalls were conducted by trained research staff over the phone within a 37-day timeframe to reduce seasonal variability in diet. The 24-h dietary recalls used a standardized multi-pass approach. Materials needed to support data collection were mailed to participants prior to administration of the surveys. Participants were compensated $110 for completing the three surveys. A more detailed description of the study protocol is provided elsewhere [19].

### Measures

#### Shopping behaviors, demographics, attitudes

Characteristics related to shopping were measured by asking participants to name the two food retailers where most of their food was purchased, the types of retailers used, frequency of food shopping, and method of transportation. Frequency of food shopping was operationalized as monthly shopping, and it was windsorized at the 95th percentile. Participants were given multiple options for method of transportation, and the variable was operationalized as shopping with their own car or not. Demographic information including sex, education level, employment status, annual household income, and race were self-reported by participants using categorical response options. Experiencing a significant life change was defined as self-report of any of the following within the past 12 months: death of a loved one, major illness, job loss, or birth of a child. Chronic disease was defined as endorsing at least one of 11 chronic diseases included on a list (i.e. heart disease, diabetes, asthma). Participants were also asked questions related to the ease, affordability, and convenience of purchasing and eating healthy foods, using a 4-point Likert scale.

#### Food security screening

The measure of food insecurity for this analysis included endorsing at least one of two items. The first, from the Behavioral Risk Factor Surveillance System(BFRSS), was “How often in the past 12 months would you say you were worried or stressed about having enough money to buy nutritious meals?” and included a 5-point Likert scale for response with answers ranging from “always” to “never” [20]. Participants were coded as endorsing this statement if they had a response of always, usually, or sometimes. The second item, which was drawn from the USDA food security screening tool and the AAP Hunger Vital Sign™ tool, asked participants to choose from 3 responses (“often true”, “sometimes true”, and “never true”) to indicate how often they felt the following statement to be true for themselves or their household over the past 12 months: “The food that (I/we) bought just didn’t last, and (I/we) didn’t have money to get more.” Participants were coded as endorsing this item if they responded often true or sometimes true. This combination of items was selected to meet the needs of the underlying community food insecurity intervention study.

#### Healthy eating index

Healthy Eating Index-2010 (HEI-2010) scores were calculated based on self-reported 24-h dietary recall data collected via the Nutrition Data System for Research. HEI-2010 scores are a measure of diet quality based on conformity to the 2010 *Dietary Guidelines for Americans* [21]. Based on the three 24-h dietary recalls, an average HEI-2010 score was calculated, with higher average scores (maximum = 100) indicating increased alignment with dietary guidelines and better diet quality.

#### Statistical analysis

Descriptive statistics (means and standard deviations for continuous variables and frequency tables for categorical variables) were computed, and t-tests and chi-squared tests were used to test for differences between participants reporting food insecurity and those in the food secure group. The results of these comparisons can be found in Table 1. Binary logistic regression was used to estimate the odds of being food insecure adjusting for the predictors listed in Table 2. The use of SNAP, rather than income, was chosen because the two are related, however, SNAP has greater practice implications given that providers can refer for SNAP, but not for an increased income. This approach concurrently estimates the effects of any listed variable on food security status while controlling for potential confounders. The food secure group was the reference group for the outcome. There was no evidence of multicollinearity in the fully adjusted models (tolerance values ranged from .74 to .98). Given the relatively small amount of missing data (6% of items across all participants were missing) and determining that data were missing at random, listwise deletion was used to limit the analytic sample to those with complete data on all variables included in the logistic regression models [22]. Analyses were conducted in 2019 using SAS, version 9.4.

**Table 1:** Sample Characteristics of Respondents by Food Security Status

Variable	Food Insecure	Food Secure	*p*-value
Sociodemographic Characteristics			
Married (%)	34 (13.5)	35 (18.4)	0.2
Black (%)	178 (70.6)	115 (60.5)	0.084
Female (%)	194 (77.0)	139 (73.2)	0.11
Education level of high school or less (%)	165 (65.5)	110 (57.9)	0.126
Employment (%)			**< 0.001**
A homemaker	8 (3.2)	1 (0.5)	
A student	2 (0.8)	2 (1.1)	
Employed for Wages	88 (34.9)	83 (43.7)	
Out of work for < 1 year	28 (11.1)	8 (4.2)	
Out of work for > 1 year	15 (6.0)	12 (6.3)	
Refused to work	0 (0.0)	3 (1.6)	
Retired	20 (7.9)	33 (17.4)	
Self-employed	10 (4.0)	8 (4.2)	
Unable to work	81 (32.1)	40 (21.1)	
Number of people living in household (mean (SD))	2.46 (1.51)	2.19 (1.30)	**0.049**
Experienced significant changes in life in past year (%)	113 (44.8)	56 (29.5)	**< 0.001**
Receives SNAP benefits (%)	184 (73.0)	104 (54.7)	**< 0.001**
Income			**< 0.001**
Less than $10,000	116 (46.0)	51 (26.8)	
$10,000–$20,000	82 (32.5)	54 (28.4)	
$20,001–$30,000	32 (12.7)	35 (18.4)	
Over $30,000	21 (8.3)	50 (26.3)	
Health Information			
Have health insurance (%)	234 (92.9)	178 (93.7)	0.839
Chronic disease (%)	138 (54.8)	86 (45.3)	0.06
Food Shopping and Dietary Behaviors			
Frequency of food shopping at primary store in days per month (mean (SD))	4.85 (5.62)	3.82 (4.17)	**0.034**
Shops with own car (%)	117 (46.4)	117 (61.6)	**0.002**
2010 Healthy Eating Index score (mean (SD))	48.73 (9.51)	51.98 (10.82)	**< 0.001**
Beliefs about Food Shopping and Diet			
I have enough time to shop for fresh and healthy foods. (%)			0.056
Strongly Agree	143 (56.7)	127 (66.8)	
Tend to Agree	81 (32.1)	54 (28.4)	
Tend to Disagree	19 (7.5)	6 (3.2)	
Strongly Disagree	9 (3.6)	3 (1.6)	
It is convenient for me to purchase fresh and healthy food. (%)			**< 0.001**
Strongly Agree	124 (49.2)	111 (58.4)	
Tend to Agree	76 (30.2)	65 (34.2)	
Tend to Disagree	33 (13.1)	13 (6.8)	
Strongly Disagree	19 (7.5)	1 (0.5)	
Eating a fresh and healthy diet is affordable (%)			**0.001**
Strongly Agree	56 (22.2)	50 (26.3)	
Tend to Agree	86 (34.1)	82 (43.2)	
Tend to Disagree	67 (26.6)	48 (25.3)	
Strongly Disagree	110 (43.7)	58 (30.5)	
It is easy to eat a fresh and healthy diet (%)			**< 0.001**
Strongly Agree	68 (27.0)	75 (39.5)	
Tend to Agree	89 (35.3)	76 (40.0)	
Tend to Disagree	62 (24.6)	32 (16.8)	
Strongly Disagree	33 (13.1)	7 (3.7)	

*Notes*: 1. Boldface *p*-values indicate statistical significance(*p* < 0.05)

2. Differences between continuous items were assessed using independent means t-tests and differences between categorical items were assessed using chi-square tests

3. Sample sizes for items differ due to missing data

**Table 2:** Multivariable Association Between Select Characteristics and Food Security Status

Variable	Estimate (SE)	OR (95% CI)
**Sociodemographic Characteristics**		
Education level of high school or less	0.315 (−0.137, 0.766)	1.37 (0.872, 2.151)
Receives SNAP benefits	**0.591 (0.129, 1.052)**	**1.805 (1.138, 2.864)**
Experienced significant changes in life in past year	**0.63 (0.196, 1.064)**	**1.877 (1.216, 2.899)**
**Health Information**		
Chronic disease	**0.462 (0.041, 0.883)**	**1.587 (1.042, 2.417)**
**Food Shopping and Dietary Behaviors**		
Store 1 shopping frequency in months	**0.099 (0.032, 0.166)**	**1.104 (1.033, 1.18)**
Shops with own car	**−0.589 (−1.038, −0.14)**	**0.555 (0.354, 0.869)**
2010 Healthy Eating Index score	−0.02 (− 0.042, 0.002)	0.98 (0.959, 1.002)
**Beliefs about Food Shopping and Diet**		
I have enough time to shop for fresh and healthy foods	−0.265 (− 0.6, 0.07)	0.767 (0.549, 1.072)
It is convenient for me to purchase fresh and healthy food	**−0.304 (− 0.605, − 0.003)**	**0.738 (0.546, 0.997)**
Eating a fresh and healthy diet is affordable	− 0.17 (− 0.425, 0.084)	0.844 (0.654, 1.088)
It is easy to eat a fresh and healthy diet	**− 0.317 (− 0.58, − 0.056)**	**0.728 (0.56, 0.946)**

*Note*: Boldface indicates statistical significance (*p* < 0.05)

## Results

### Sample characteristics

The study population included 442 people. Most participants were female (75.9%) and unmarried (84.4%). The average age of respondents was 51 years old (SD = 13.2 years), and 67.5% of the total respondents were Black. The average household size was slightly less than the national average in 2015(2.5 people per household), at 2.46 people in the food insecure group, and 2.19 people in the food secure group. Nearly two-thirds of the respondents (62.2%) reported having a high school education or less, and less than 39% of respondents were employed for wages. Nearly all respondents (95.2%) had health insurance at the time of survey data collection.

### Demographics and attitudes

A summary of participant characteristics delineated by food insecurity status is included in Table 1. Participants were considered part of the food insecure group if they endorsed at least one of the two food insecurity questions, and in the food secure group if they did not endorse either item. Over half of the respondents (57%) answered affirmatively to at least one of the two food insecurity screening items. These participants (*n* = 252) were considered food insecure for this analysis. Those in the food insecure group were more likely to report having a chronic illness, though only marginally significant (*p* = 0.06), and significantly less likely to describe a fresh healthy diet as “easy”, “affordable,” or “convenient” (*p* < 0.001).

### Diet and food behaviors

Those in the food insecure group had significantly lower HEI-2010 scores (*p* < 0.001), indicating a lower overall diet quality. Most in the food insecure group (73%) received SNAP benefits. Food insecure participants shopped with significantly more frequency (4.9 days per month) than their counterparts who were categorized as food secure (3.8 days per month) (*p* < 0.05). Despite shopping more frequently, fewer food insecure participants utilized their own car for food shopping than those who were food secure (46.4% compared to 61.6%, respectively) (*p* = 0.002).

### Multivariable analyses

Using a logistic regression model, we estimated the association between several variables and the food insecure classification based on the two-item screener. Table 2 displays the odds ratios and associated *p*-values for 11 characteristics included in the model. Affirmative attitudes related to convenience of shopping for healthy foods [OR = 0.74; 95% CI = (0.55,1.00)] and ease of eating healthy foods [OR = 0.73; 95% CI = (0.56, 0.95)] were associated with decreased odds of reporting food insecurity. Similarly, those that used their own car when shopping for food had lower odds of reporting food insecurity [OR = 0.55; 95% CI = (0.35, 0.87)].

Compared to those who did not report having any chronic disease, among those with a with a chronic disease, we see a 59% increase in the odds of food insecurity [OR = 1.59; 95% CI = (1.04, 2.42)]. As shopping frequency increased, the odds of food insecurity also increased [OR = 1.10; 95% CI = (1.03, 1.18)]. The odds of reporting food insecurity also increased when participants experienced a significant life event within the past 12 months [OR = 1.88; 95% CI = (1.22,2.90)]. Participants that received SNAP benefits are expected to have an 81% increase in the odds of reporting food insecurity, compared to those not receiving SNAP benefits [OR = 1.81; 95% CI = 1.14, 2.86)]. HEI-2010 scores, education, and perceptions of affordability and time associated with healthy foods did not produce statistically significant results.

## Discussion

This cross-sectional survey found that over half of the participants experienced food insecurity. Study participants were from households located in two midwestern urban neighborhoods with low access to healthy food retailers. Our finding that those with chronic conditions have higher odds of food insecurity underscores the clinical relevance of brief screening. These results also highlight the fact that universal clinic-based screening by providers serving these communities may identify high levels of need beyond food resources, which may present new challenges and opportunities.

One of the most common actions taken in response to a positive screen for food insecurity in the clinical setting is referral to Supplemental Nutrition Assistance Program (SNAP) [17, 23]. The fact that nearly three-quarters of the households experiencing food insecurity in our survey were already receiving SNAP suggests that this approach is necessary but not always sufficient for meeting food needs. Residual food insecurity in the SNAP population has been linked to factors including negative income shocks or changes in household composition [24]. It might also be driven by associations with other non-random correlates of SNAP participation [25]. Other supplemental low cost or no cost food sources, including community-based food pantries and nutrition incentive programs like *Double Up Food Bucks* [26, 27], should be included in the food insecurity referral model.

Another explanation for this finding is that SNAP benefits may not surmount food access barriers beyond costs. Those who described a fresh and healthy diet as affordable, easy, or convenient had lower odds of screening positive for food insecurity. These attitudes related to ease or convenience may align with food insecure respondents’ greater number of shopping trips and the greater need to use transportation other than their own car. Other research on shopping patterns has shown that food insecure families travel shorter distances to complete their grocery shopping [28], which may indicate transportation barriers as limitations to shopping in stores beyond that of corner or convenience stores that tend to offer fewer healthy options. However, findings on the type of retailers that food insecure individuals frequent for food purchasing have been mixed [12, 13, 28]. As such, responses to food insecurity needs may be most successful when they include instrumental supports (e.g., bus passes, taxi vouchers, food delivery, ready-to-eat meal kits) that help people work around barriers, and motivational interviewing to support changes in attitudes toward and perceptions of healthy eating and food shopping behaviors. Supports to reduce the transportation barrier related to food access also has implications for access to health care and other services that may otherwise be limited by lack of transportation. Findings emphasize the value of clinical-community linkages that extend healthcare services to include strategic connections with community services. This may include connections to food-related services such as food pantries, nutrition education and navigation [29], nutrition incentive programs like *Double Up Food Bucks*, community gardens, and food stores that offer healthy foods at affordable prices as well as non-food related services such as transportation and financial supports [30]. The common approach of providing a list of resources related to social needs without facilitation of accessing these resources is likely not enough to generate lasting improvement [17].

It is important to recognize that food insecurity does not exist in a vacuum and may be a “symptom” of larger socioeconomic struggles. Consistent with previous research, our analysis found that significant life events, such as death of a loved one, legal troubles, change in finances, or job loss were nearly twice as common in those experiencing food insecurity [31]. The cross-sectional nature of these data does not allow us to determine the direction of causation, but food insecurity is likely an indication of many needs, beyond just food [32]. Given the limited ability to control some of these life changes, resources related to coping or perhaps alleviating some of the burden that these events put on families may be feasible ways to prevent food insecurity among patients and their families.

Based on the current analysis, we argue that the value of brief screening for food security in primary care starts with needs identification and referral yet expands to included considerations related to strategically connecting patient needs with both food and non-food related supports. These screenings reveal much more than worry about food. Accordingly, they provide a tool for tailoring health care delivery to effectively support patients or families in higher need of social, financial, and community resources.

### Limitations

First, this study used two independent validated food insecurity items rather than a validated scale like the Hunger Vital Sign™ tool. This measurement approach was selected to meet the needs of the primary study from which this data is drawn. As such, the results do not fully generalize to the food insecure populations generated from more standardized measurement approaches. Secondly, the cross-sectional nature of the study limits us to discussion of associations rather than causation. Next, given that food security can be transient, an individual could endorse at least one of the given questions at the time of interview, perhaps if they were feeling the effects more strongly at that point, and fluctuate throughout the year. However, the US Economic Research Service (ERS) reports that many households who experience food insecurity at some point in the year experienced this for 7 months during the year, suggesting that although perhaps transient, food insecurity can be persistent [1]. Thus, there may still be benefit in understanding that individuals have experienced food insecurity within the year, coupled with additional barriers or lack of resources.

The measure of food insecurity applied in this research included the use of two separately validated, nationally used items, rather than the use of one tool. Surveys utilized in this study were developed prior to the wide adoption and standardization of the two-item Hunger Vital Sign™. Finally, results provide insight into characteristics associated with food insecurity among a community sample rather than a clinical population. However, half of the study population reported a chronic illness and over 90% are insured.

## Conclusions

Food insecurity is likely a common SDH among patients living in low-income urban neighborhoods with low access to grocery stores. In addition to screening for food insecurity within health care settings, findings offer suggestions for the types of community-clinical linkages that may need to be fostered to effectively treat patients experiencing food insecurity and potentially lacking other resources related to SDH. Findings suggest effective referral mechanisms to both food and non-food related community services are warranted within health care systems to modulate SDH and the effect they have on health outcomes. The available research related to SDH screening points to the importance of understanding characteristics and needs of patients for them to achieve the best health outcomes. Seamless connections linking patients to community resources—allowing for feedback within the health care setting—will be needed to optimize public health impact.

## Supplementary Information

**Additional file 1.** Foodnest year 2 survey: food shopping, food access, nutrition & health.

## Data Availability

The datasets used and/or analyzed during the current study are available from the corresponding author on reasonable request.

## Abbreviations

SDH: Social determinants of health
USDA: US Department of Agriculture
AAP: American Academy of Pediatrics
SNAP: Supplemental Nutrition Assistance Program
HEI-2010: Healthy Eating Index-2010
US ERS: US Economic Research Service
