# Supplementary material for: Identification of factors related to food insecurity and the implications for social determinants of health screenings
Source: BMC Public Health. 2021 Jul 16;21:1410. doi: 10.1186/s12889-021-11465-6 (PMC8284017; doi:10.1186/s12889-021-11465-6)
Supplement: Supplementary file 1 — Additional file 1. Foodnest year 2 survey: food shopping, food access, nutrition & health. [file 12889_2021_11465_MOESM1_ESM.pdf]

Future of Food in Your Neighborhood Study
Co-PI: Darcy Freedman, PhD, MPH
Co-PI: Ashwini Sehgal, MD
Grant Number: R01DK108184
For more information, contact us at foodNEST@case.edu or (216) 368-7578

foodNEST YEAR 2 SURVEY: FOOD SHOPPING, FOOD ACCESS, NUTRITION & HEALTH

INTERVIEWER: I appreciate your time and patience so far. I am now going to shift the focus of the questions to learn more about your food shopping habits, food access, neighborhood opinions, and nutrition and health. These questions will take about 30 minutes or less to complete. Then we are done for today. As a reminder, after you complete this survey, we will send you your $35 gift card.

There are no right or wrong answers to these questions. You can decline to answer any of the questions. It is most important to provide your honest thoughts and opinions.

Occasionally, I will ask you to refer to page 20 of your Food Amounts Booklet, so please keep it within reach.

INTERVIEWER: Many of the questions will focus on the past 12 months. That would be since your first interview with foodNEST on [SURVEY 1 DATE].

INTERVIEWER: I will first be asking you questions about your food shopping and food preferences.

1. How involved were you in <u>food shopping</u> for your household over the last 12 months. Would you say that you were…
   ☐ 1 Involved in about half of the food shopping
   ☐ 2 Involved in more than half of the food shopping
   ☐ 3 Involved in all of the food shopping
   ☐ 4 None of the above *(do not read)*

2. How involved were you in <u>preparing meals</u> for your household over the last 12 months. Would you say that you were…
   ☐ 1 Not involved at all in meal preparation
   ☐ 2 Involved in less than half of meal preparation
   ☐ 3 Involved in about half of meal preparation
   ☐ 4 Involved in more than half of meal preparation
   ☐ 5 Involved in all of meal preparation

INTERVIEWER: These next questions are about where you shop for food.

3. Over the past 12 months, about how much of your household food shopping would you say is done within a 20 minute walk or about a mile from your home? Would you say…
   ☐ 1 None or almost none of it
   ☐ 2 Some of it
   ☐ 3 About half of it
   ☐ 4 Most of it
   ☐ 5 All or almost all of it

Do not read:
☐ 777 Don’t know / Not sure

☐ 999 Refused

4. Please tell me all the places where you got food for your household over the past 12 months. I will list different options. Did you shop for or receive food from …

- ☐ 1 Supermarket (e.g. Dave's, Kroger)
- ☐ 2 Supercenter (e.g. Wal-Mart, Target)
- ☐ 3 Smaller grocery stores (Marc's, Aldi's, Save a Lot, IGA, Sheliga)
- ☐ 4 Warehouse club (Sam's Club, BJs, Costco)
- ☐ 5 Convenience store/Corner store
- ☐ 6 Specialty store (e.g. ethnic stores, meat market, seafood market, green grocer, bakery)
- ☐ 7 Drug/Pharmacy (e.g. CVS, Rite Aid, Walgreens)
- ☐ 8 Dollar variety (Dollar General, Dollar Tree)
- ☐ 9 Farmers' market (Mae's Produce, St. Clair Farmers' Market)
- ☐ 10 Food pantry/bank or shelter (Hunger Network)
- ☐ 11 Mobile Pantry/Produce Drop Off
- ☐ 12 Church
- ☐ 13 Friends or family
- ☐ 14 Other, specify____
- ☐ 777 Don't know

5. How frequently did you visit [SELECTION 1-13] over the past 12 months to get food for your household? You can answer in the amounts per day, week, month, or year.

____1 times per day	____2 times per week	____3 times per month	____4 times per year

5a. Number of times:__________________________

6. What are the names of the two stores where you bought most of the food for your household over the past 12 months? Please start with the one where you do the most of your shopping.
   6a. Record name of main store (Store 1): ________________________
   6b. Record name of secondary store (Store 2): ______________________

INTERVIEWER: Now I will ask you a few questions about both stores separately.

7. Please tell me the street name and city where [STORE 1] is located?

   Record Street Name: ________________________

Record City: ________________________________
Don't know
If "Don't know" is chosen, ask "Could you give me the cross streets, nearest major intersection, or neighborhood where [STORE 1] is located? ____

NOTE: Ask only if necessary.

8. What kind of food store is this?

Store 1
☐ 1 Supermarket (e.g. Giant Eagle, Kroger, Daves)
☐ 2 Supercenter (e.g. Wal-Mart, Target)
☐ 3 Smaller grocery stores (e.g. Aldi, Save a Lot, IGA, Sheliga)
☐ 4 Warehouse club (e.g. Sam's Club, BJs, Costco)
☐ 5 Convenience store (with or without a gas station attached)
☐ 6 Specialty store (e.g. ethnic specialty stores, meat market, seafood market, green grocer, bakery)
☐ 7 Drug/Pharmacy (e.g. CVS, Rite Aid, Walgreens)
☐ 8 Dollar variety (e.g. Dollar General, Dollar Store, Family Dollar)
☐ 9 Farmers' market (St. Clair Farmers' Market, Mae's Produce)
☐ 10 Food pantry or shelter
☐ 11 Other
☐ 777 Don't Know

9. Over the past 12 months, about how much of your household food shopping would you say is done at [STORE 1]? Would you say….
   - ☐ 1 Almost none of it
   - ☐ 2 Some of it
   - ☐ 3 About half of it
   - ☐ 4 Most of it
   - ☐ 5 All or almost all of it

Do not read:
- ☐ 777 Don't know / Not sure
- ☐ 999 Refused

10. Over the past 12 months, what mode of transportation did you use most often for your shopping trip to [STORE 1]? *Check all that apply.* (If participant says "car" make sure to clarify their own person car, or someone else's car.)
    - ☐ 1 Personal vehicle
    - ☐ 2 Ride in car/van/truck of family or friends
    - ☐ 3 Public transportation
    - ☐ 4 Taxi/Uber
    - ☐ 5 Walk or bike

Do not read:

- ☐ 777 Don't know / Not sure
- ☐ 999 Refused

11. How frequently did you visit [STORE 1] over the last 12 months? [Interview note: Pick one timeframe that best represents their pattern and record number.]

____ 1 times per day	____ 2 times per week	____ 3 times per month	____4 times per year

11a. Number of times:____________________________

12. Which day of the week do you typically go to [STORE 1]? *Choose one.*
    - ☐ 1 Sunday
    - ☐ 2 Monday
    - ☐ 3 Tuesday
    - ☐ 4 Wednesday
    - ☐ 5 Thursday
    - ☐ 6 Friday
    - ☐ 7 Saturday
13. What time of day do you typically go to [STORE 1]? *Choose one.*
    - ☐ 1 Mornings (7am – 10am)
    - ☐ 2 Midday (11am – 3pm)
    - ☐ 3 Evenings (4pm – 7pm)
    - ☐ 4 Night (8pm – midnight)

14. Do you use store deals, sales, or coupons at [STORE 1]?
    - ☐ 1 Yes
    - ☐ 2 No
    - ☐ 777 DK
    - ☐ 999 Refuse

15. Do you use a store loyalty card or a frequent shopper card at [STORE 1]?
    - ☐ 1 Yes
    - ☐ 2 No
    - ☐ 777 DK
    - ☐ 999 Refuse

16. How do you usually pay at [STORE 1]? *Check all that apply [INTERVIEW NOTE: Do not read answer choices]*
    - ☐ 1 Cash
    - ☐ 2 Check
    - ☐ 3 WIC
    - ☐ 4 SNAP/EBT

- ☐ 5 Debit Card
- ☐ 6 Credit Card
- ☐ 7 TANF
- ☐ 8 Other, *please specify:*___________________________________
- ☐ 777 DK
- ☐ 999 Refuse

INTERVIEWER: Now I will move forward to asking the same questions for [STORE 2].

NOTE: Ask only if necessary

17. What kind of food store is [STORE 2]?

**Store 2**
☐ 1 Supermarket (e.g. Giant Eagle, Kroger, Daves)
☐ 2 Supercenter (e.g. Wal-Mart, Target)
☐ 3 Smaller grocery stores (e.g. Aldi, Save a Lot, IGA, Sheliga)
☐ 4 Warehouse club (e.g. Sam's Club, BJs, Costco)
☐ 5 Convenience store (with or without a gas station attached)
☐ 6 Specialty store (e.g. ethnic specialty stores, meat market, seafood market, green grocer, bakery)
☐ 7 Drug/Pharmacy (e.g. CVS, Rite Aid, Walgreens)
☐ 8 Dollar variety (e.g. Dollar General, Dollar Store, Family Dollar)
☐ 9 Farmers' market (e.g. St. Clair Famers' Market, Mae's Produce)
☐ 10 Food pantry or shelter
☐ 11 Other
☐ 777 Don't Know

18. Over the past 12 months, about how much of your household food shopping would you say is done at [STORE 2]? Would you say…
    - ☐ 1 Almost none of it
    - ☐ 2 Some of it
    - ☐ 3 About half of it
    - ☐ 4 Most of it
    - ☐ 5 All or almost all of it

Do not read:

- ☐ 777 Don't know / Not sure
- ☐ 999 Refused

19. Over the past 12 months, what mode of transportation did you use most often for your shopping trip to [STORE 2]? *Check all that apply.* (If participant says "car" make sure to clarify their own person car, or someone else's car.)
    - ☐ 1 Personal vehicle
    - ☐ 2 Ride in car/van/truck of family or friends

- ☐ 3 Public transportation
- ☐ 4 Taxi/Uber
- ☐ 5 Walk or bike

Do not read:

- ☐ 777 Don't know / Not sure
- ☐ 999 Refused

20. How frequently did you visit [STORE 2] over the last 12 months? [Interview note: Pick one timeframe that best represents their pattern and record number.]

____ times per day	____ times per week	____ times per month	____ times per year

20a. Number of times:________________________

21. Which day of the week do you typically go to [STORE 2]? *Choose one.*
- ☐ 1 Sunday
- ☐ 2 Monday
- ☐ 3 Tuesday
- ☐ 4 Wednesday
- ☐ 5 Thursday
- ☐ 6 Friday
- ☐ 7 Saturday

22. What time of day do you typically go to [STORE 2]? *Choose one.*
- ☐ 1 Mornings (7am – 10am)
- ☐ 2 Midday (11am – 3pm)
- ☐ 3 Evenings (4pm – 7pm)
- ☐ 4 Night (8pm – midnight)

23. Do you use store deals, sales, or coupons at [STORE 2]?
- ☐ 1 Yes
- ☐ 2 No
- ☐ 777 DK
- ☐ 999 Refuse

24. Do you use a store loyalty card or a frequent shopper card at [STORE 2]?
- ☐ 1 Yes
- ☐ 2 No
- ☐ 777 DK
- ☐ 999 Refuse

25. How do you usually pay at [STORE 2]? *Check all that apply. [INTERVIEW NOTE: Do not read answer choices]*

- ☐ 1 Cash
- ☐ 2 Check
- ☐ 3 WIC
- ☐ 4 SNAP/EBT
- ☐ 5 Debit Card
- ☐ 6 Credit Card
- ☐ 7 TANF
- ☐ 8 Other, please specify:____
- ☐ 777 DK
- ☐ 999 Refuse

INTERVIEWER: The next questions are about other food outlets you may use.

26. Over the past 12 months, how often did you buy food at a restaurant, fast food restaurant, or for carry-out?

Read only if necessary:

- ☐ 1 Never
- ☐ 2 A few times a year
- ☐ 3 Monthly
- ☐ 4 Every 2 weeks
- ☐ 5 Weekly
- ☐ 6 Two or more times a week
- ☐ 7 At least once a day

Do not read:

- ☐ 777 Don't know / Not sure
- ☐ 999 Refused

27. Over the past 12 months, how often did you buy food at a farmers' market or produce stand during the summer and fall seasons?

Read only if necessary:

- ☐ 1 Never
- ☐ 2 A few times a year
- ☐ 3 Monthly
- ☐ 4 Every 2 weeks
- ☐ 5 Weekly
- ☐ 6 Two or more times a week
- ☐ 7 At least once a day

Do not read:

- ☐ 777 Don't know / Not sure
- ☐ 999 Refused

28. Over the past 12 months, did anyone in your household have any of the following dietary restrictions that you took into account during your food shopping for the household? Please choose all the following that apply…

- ☐ 1 Vegetarian or vegan
- ☐ 2 Lactose intolerant

- ☐ 3 Food allergies
- ☐ 4 Dieting for weight loss
- ☐ 5 Low-sodium
- ☐ 6 Low-potassium
- ☐ 7 Gluten-free
- ☐ 8 Low-sugar
- ☐ 9 Low –fat
- ☐ 10 Low-carb
- ☐ 11 Other, specify____
- ☐ 12 None (skip to Q30)

28a. Specify other____

INTERVIEWER: For some of the next questions we ask you think about your neighborhood. For this, please think about your neighborhood as the area within a 20 minute walk or 1 mile from your home.

29. In your neighborhood, how easy or difficult is it to buy foods needed for the dietary restrictions for you or others in your household? Would you say it is...
   - ☐ 1 Very difficult
   - ☐ 2 Difficult
   - ☐ 3 Easy
   - ☐ 4 Very Easy

Do not read:

- ☐ 777 Don't know / Not sure
- ☐ 999 Refused

Please turn to response chart D in your food amounts booklet. Please tell me how you would rate the following questions from not at all interested to very interested.

If participant does not have response card, say the following: Your responses are not at all interested, somewhat interested, very interested.

30. How interested would you be in buying food from an online food delivery service that would bring groceries to a central drop off place in your neighborhood where you would go to pick up your food delivery?
   - ☐ 1 Not at all interested [SKIP to Q32]
   - ☐ 2 Somewhat interested
   - ☐ 3 Very interested

31. What type of central drop off location would be best in your neighborhood? Check all that apply. [INTERVIEWER NOTE: Read full list]
   - ☐ 1 Church or Faith based organization
   - ☐ 2 School
   - ☐ 3 Daycare
   - ☐ 4 Community center

- ☐ 5 Corner store
- ☐ 6 Other, specify_____________________________________

32. How interested would you be in buying food from an online food delivery service that would bring groceries to your home?
    - ☐ 1 Not at all interested
    - ☐ 2 Somewhat interested
    - ☐ 3 Very interested

33. How do you get on the internet most often? Select one option. [INTERVIEWER NOTE: Do not read list]
    - ☐ 1 Personal computer at home
    - ☐ 2 Computer at a friend or family member's home
    - ☐ 3 Cell phone
    - ☐ 4 Library
    - ☐ 5 School
    - ☐ 6 Community center
    - ☐ 7 Work
    - ☐ 8 Other, specify:____
    - ☐ 9 I do not get on the internet

INTERVIEWER: Please turn to the "response chart" onpage 20 of your Food Amounts Booklet. For this set of questions, please refer to Section A.

If participant does not have response card say the following: Your answer choices are strongly disagree, tend to disagree, tend to agree, or strongly agree.

34. How much do you agree with the following statements about your neighborhood over the past 12 months? Again, think about your neighborhood as the area within a 20 minute walk or 1 mile from your home.

	**Strongly Disagree**	**Tend to disagree**	**Tend to agree**	**Strongly Agree**	**Don't Know**
a. A large selection of fruits and vegetables is available in your neighborhood.	1	2	3	4	777
b. The fresh fruits and vegetables in your neighborhood are of high quality.	1	2	3	4	777
c. A large selection of low-fat products is available in your neighborhood.	1	2	3	4	777

35. Over the last 12 months, did you take part in any of the following activities in **<u>your neighborhood</u>**?

    NOTE: Read all and check all that apply: Please confirm that any activities selected occurred in the participant's neighborhood. (Make sure to confirm that the activity occurred in THEIR neighborhood)
    - ☐ 1 Nutrition or healthy eating class (with cooking) (e.g. Vel Scott's Healthy Cooking Class, Local Matters)
    - ☐ 2 Nutrition or healthy eating class (without cooking)
    - ☐ 3 Weight loss class or program (e.g., weight watchers)

- ☐ 4 Exercise class or program
- ☐ 5 Community gardening
- ☐ 6 Home gardening
- ☐ 7 Community arts event (e.g., murals, art club, public art exhibit)
- ☐ 8 Neighborhood meetings (e.g., Ward meetings, block club, civic associations, neighborhood watch)
- ☐ 9 Neighborhood events (e.g., cookouts, picnics, festivals)
- ☐ 10 Farmers' market
- ☐ 11 Healthy shopping tour
- ☐ 12 Cooking Circles
- ☐ 13 Healthy food incentive program (e.g., Produce Perks, Veggie SNAPS, Produce Prescription)

36. Over the last 12 months, did you take part in any other activities in **your neighborhood** besides the ones just mentioned?
    - ☐ 1-Yes
    - ☐ 2-No

    34a. If yes, can you tell me a little more about these? ____

Do not read:

- ☐ 777 Don't know / Not sure
- ☐ 999 Refused

37. [SKIP IF Q35 IS BLANK and 36=2] Why did you choose to attend these neighborhood events? [INTERVIEWER NOTE: Do not read answer choices]
    - ☐ 1 Advertisement for event
    - ☐ 2 Easy to get to
    - ☐ 3 Location of Event
    - ☐ 4 Time of Event
    - ☐ 5 Knew other people attending event
    - ☐ 6 Opportunity to meet new people
    - ☐ 7 Aligned with health concerns
    - ☐ 8 Health care provider recommendation
    - ☐ 9 Interested in topic
    - ☐ 10 Invited by friend, neighbor, family
    - ☐ 11 Invited by community ambassador
    - ☐ 12 Other, specify: _______________________________________
    - ☐ 777 Don't know/unsure
    - ☐ 999 Refused

38. [SKIP IF Q35=1-13 OR Q36=1] Why did you choose not to attend these neighborhood events? [INTERVIEWER NOTE: Do not read answer choices]
    - ☐ 1 Disabled
    - ☐ 2 Schedule Conflict
    - ☐ 3 Location of Event
    - ☐ 4 Time of Event
    - ☐ 5 Didn't See Advertisement for Event

- ☐ 6 Weather
- ☐ 7 Health Problems
- ☐ 8 Did not know anyone attending event
- ☐ 9 Did not know event was happening in community
- ☐ 10 Not interested
- ☐ 11 Other, specify:____
- ☐ 777 Don't know
- ☐ 999 Refused

INTERVIEWER: For the next questions, please think about fresh and healthy foods. For this survey, when I say "fresh and healthy foods" I am referring to foods like fresh fruits and vegetables, fresh meats and dairy, and unprocessed foods.

39. Please turn to page 20 of your Food Amounts Booklet and look at the pictures at the bottom of the page. This is a sign for the Good Food Here [Cleveland] / Fresh Food Here and Veggie SNAPS [Columbus] program. To what extent did these signs influence your decision to purchase fresh and healthy foods at a store in your neighborhood? Would you say it influenced your decision…

- ☐ 1 Not at all
- ☐ 2 Slightly
- ☐ 3 Moderately
- ☐ 4 Very Much
- ☐ 5 I have not seen the signs
- ☐ 777 Don't know/not sure

40. [IF Q39 IS 1-4] Can you tell me why you selected [Q38 Choice]?

____

41. [IF Q39 IS 1-4] Where did you see these signs? [INTERVIEW NOTE: Do not read list]
    - ☐ IGA
    - ☐ Mae's Produce
    - ☐ Sheliga
    - ☐ Other (specify)________________________________

The next questions are about food shopping and cooking. Please think about the last 12 months when answering these questions.

42. How much do you agree or disagree with the following statements? Please refer to Section A of your response chart for your response choices. *[Interviewer Note: Do not say 'don't know.']*

    If participant does not have response card say the following: Your answer choices are strongly disagree, tend to disagree, tend to agree, or strongly agree.

	Strongly Disagree	Tend to Disagree	Tend to Agree	Strongly Agree	Don't Know
a. I have enough time to shop for fresh and healthy foods.	1	2	3	4	777
b. It is convenient for me to purchase fresh and healthy foods	1	2	3	4	777
c. I think that fresh and healthy foods are expensive.	1	2	3	4	777

***INTERVIEWER: Now I'm going to ask you some questions about your family, neighbors, and other people you interact with.* Please refer to Section B of your response chart for your response choices.**

If participant does not have response card, say the following: Your answer choices are never, hardly ever, now and again, quite often, and most of the time.

43. How often during the past 12 months have members of your family…

Item	Never	Hardly ever	Now and Again	Quite Often	Most of the time	Don't know
a. Eaten fresh and healthy foods with you	1	2	3	4	5	777
b. Encouraged you to eat fresh and healthy foods	1	2	3	4	5	777
c. Discouraged you from eating unhealthy foods	1	2	3	4	5	777
d. Told you about fresh and healthy foods and how to prepare them	1	2	3	4	5	777
e. Prepared fresh and healthy foods with you	1	2	3	4	5	777

44. How often during the past 12 months have neighbors…

Item	Never	Hardly ever	Now and Again	Quite Often	Most of the time	Don't know
a. Eaten fresh and healthy foods with you	1	2	3	4	5	777
b. Encouraged you to eat fresh and healthy foods	1	2	3	4	5	777

c. Discouraged you from eating unhealthy foods	1	2	3	4	5	777
d. Told you about fresh and healthy foods and how to prepare them	1	2	3	4	5	777
e. Prepared fresh and healthy foods with you	1	2	3	4	5	777

INTERVIEWER: For the next set of questions, please refer to Section A of your response chart for your response choices.

If participant does not have response card, say the following: Your answer choices are strongly disagree, tend to disagree, tend to agree, or strongly agree.

45. How much do you agree or disagree with the following statements? Again, think about the last 12 months when answering these questions.

	**Strongly Disagree**	**Tend to Disagree**	**Tend to Agree**	**Strongly Agree**	**Don't Know**
a. Most people who are close to you think it is important to buy fresh and healthy foods.	1	2	3	4	777
b. Your family expects you to buy fresh and healthy foods.	1	2	3	4	777
c. Your neighbors expect you to buy fresh and healthy foods.	1	2	3	4	777

INTERVIEWER: The next questions are about your diet over the past 12 months. Please refer to Section A of your response chart for your response choices.

If participant does not have response card, say the following: Your answer choices are strongly disagree, tend to disagree, tend to agree, or strongly agree.

46. How much do you agree or disagree with the following statements

	**Strongly Disagree**	**Tend to Disagree**	**Tend to Agree**	**Strongly Agree**	**Don't Know**

a. I am a healthy eater.	1	2	3	4	777
b. I am someone who eats in a nutritious manner.	1	2	3	4	777
c. I am someone who is careful about what I eat.	1	2	3	4	777

INTERVIEWER: For the next set of questions, please refer to Section A of your response chart for your response choices. Again, when I say "fresh and healthy foods" in these questions, I am referring to foods like fresh fruits and vegetables, fresh meats and dairy, and unprocessed foods.

If participant does not have response card, say the following: Your answer choices are strongly disagree, tend to disagree, tend to agree, or strongly agree.

47. How much do you agree or disagree with the following statements based on your experiences over the past 12 months?

	**Strongly Disagree**	**Tend to Disagree**	**Tend to Agree**	**Strongly Agree**	**Don't Know**
a. Eating a fresh and healthy diet tastes bad.	1	2	3	4	777
b. Eating a fresh and healthy diet is affordable.	1	2	3	4	777
c. It is easy to eat a fresh and healthy diet	1	2	3	4	777
d. I don't need to change my diet as it is healthy enough	1	2	3	4	777

INTERVIEWER: Now I'd like for you to refer to Section B of your response chart for your response choices.

If participant does not have response card, say the following: Your answer choices are hardly ever, now and again, quite often, or most of the time.

48. Over the past 12 months, how often did you…

	Never	Hardly ever	Now and Again	Quite Often	Most of the time
a. Make conscious efforts to try and eat a fresh and healthy diet?	1	2	3	4	5
b. Try to keep the amount of fat you eat to a healthy amount?	1	2	3	4	5
c. Try to keep the amount of sugar you eat to a healthy amount?	1	2	3	4	5

---ASK OF CLEVELAND PARTICIPANTS ONLY--

INTERVIEWER: The next questions are focused on your thoughts about Hub 55 on 55th and St. Clair which opened in your neighborhood. Hub 55 is made up of a farmers' market (St. Clair Farmers' Market), a healthy eating café (Café 55), and a brewery (Goldhorn Brewery). I will ask you questions about each of these places. First I will ask you questions about working at these places.

49. Over the past 12 months, have you worked at or been a vendor at any of the following places: St. Clair Farmers' Market, Café 55, or Goldhorn Brewery?

☐ 1 Yes
☐ 2 No [SKIP TO Q50]

49aIf yes, please specify where:_

☐ 1 St. Clair Farmers' Market
☐ 2 Café 55
☐ 3 Goldhorn Brewery

Now I will ask you questions about visiting the farmers' market.

50. Over the past 12 months, how frequently did you visit the St. Clair Farmers' Market at Hub 55 on 55th and St. Clair? [Interview note: Pick one timeframe that best represents their pattern and record number.].

____ 1 times per day	____ 2 times per week	____ 3 times per month	____4 times per year	___5 Never visited [SKIP TO Q54]

50a. Number of times:____________________________

51. [IF Q50 = 1-4] What are the two best things about the St. Clair Farmers' Market? [INTERVIEW NOTE: Write "DK" if participant has no response]

1. ____
2. ____

52. [IF Q50 = 1-4] What are two things that need to be improved at St. Clair Farmers' Market? [INTERVIEW NOTE: Write "DK" if participant has no response]
1. ____
2. ____

53. [IF Q50 = 1-4] During your visits to the St. Clair Farmers' Market over the past 12 months, what types of food did you buy? *Check all that apply. INTERVIEWER NOTE: Do NOT read full list.*

☐ 1 Fresh fruits
☐ 2 Fresh vegetables
☐ 3 Fresh meat
☐ 4 Fish or seafood
☐ 5 Cheese or dairy
☐ 6 Bakery (e.g. breads, pastries)
☐ 7 Pasta and dry goods (e.g. beans, grains)
☐ 8 Prepared or ready-to-eat food (e.g. pierogis, pizza, tacos)
☐ 9 Value-added foods (e.g. pre-cut fruits and vegetables, salsa, jams/jelly)
☐ 10 Staples (e.g., coffee, flour, sugar)
☐ 11 General convenience items (e.g. soap)
☐ 12 Herbs and spices
☐ 13 Fresh cut flowers or plants
☐ 14 Other, specify:_______________________________________)

54. [IF Q50 =5] Why didn't you visit the St. Clair Farmers' Market during the past 12 months? *INTERVIEWER NOTE: Do NOT read full list.*

☐ 1 Disabled
☐ 2 Schedule conflict
☐ 3 Location of market
☐ 4 Time of market
☐ 5 Did not know market was happening in community
☐ 6 Weather
☐ 7 Health problems
☐ 8 Did not know anyone attending market
☐ 9 Not interested
☐ 10 Don't like what it sells
☐ 11 This place is not for me
☐ 12 Too expensive
☐ 13 Did not feel welcome there
☐ 14 Other, specify:__________________________________
☐ 777 Don't know/unsure
☐ 999 Refused

For the next questions, I'd like your opinions about the St. Clair Farmers' Market at Hub 55 on 55th and St. Clair. This can be based on your experience or on what others have said about the St. Clair Farmers' Market.

Please turn to response chart C in your food amounts booklet. Please tell me how you would rate the following questions from poor to excellent.

If participant does not have response card, say the following: Your responses are poor, fair, good, very good, or excellent.

55. How would you rate the quality of foods at the St. Clair farmers' market?

☐ 1 Poor
☐ 2 Fair
☐ 3 Good
☐ 4 Very Good
☐ 5 Excellent
☐ 777 Don't know/unsure

56. How would you rate the prices of foods at the St. Clair farmers' market?

☐ 1 Poor
☐ 2 Fair
☐ 3 Good
☐ 4 Very Good
☐ 5 Excellent
☐ 777 Don't know/unsure

Now I will ask you questions about visiting Café 55.

57. Over the past 12 months, how frequently did you visit Café 55 at Hub 55 on 55th and St. Clair? [Interview note: Pick one timeframe that best represents their pattern and record number.]

_____ 1 times per day	_____ 2 times per week	_____ 3 times per month	_____4 times per year	___5 Never visited [SKIP TO Q61]

57a. Number of times:____________________________

58. [IF Q57 IS 1-4] What are the two best things about Café 55? [INTERVIEW NOTE: Write "DK" if participant has no response]

1. ____
2. ____

59. [IF Q57 IS 1-4] What are two things that need to be improved at Café 55? [INTERVIEW NOTE: Write "DK" if participant has no response]

1. ____
2. ____

60. [IF Q57 IS 1-4] During your visits to Café 55 over the past 12 months, what types of food did you buy? *Check all that apply*? *Check all that apply. INTERVIEWER NOTE: Do NOT read full list.*

☐ 1 Breakfast bowl
☐ 2 Lunch bowl
☐ 3 Dessert/pastry
☐ 4 Snack (chips, bagels)
☐ 5 Beverage (non-alcoholic)
☐ 6 Beverage (alcoholic)
☐ 7 Other (specify: ______________________________________)

61. [IF Q57 IS 5] Why didn't you visit Café 55 during the past 12 months? [Check all that apply] *INTERVIEWER NOTE: Do NOT read full list.*

☐ 1 Disabled
☐ 2 Schedule conflict
☐ 3 Location of café
☐ 4 Time of café
☐ 5 Did not know café was happening in community
☐ 6 Weather
☐ 7 Health problems
☐ 8 Did not know anyone attending café
☐ 9 Not interested
☐ 10 Don't like what it sells
☐ 11 This place is not for me
☐ 12 Too expensive
☐ 13 Did not feel welcome there
☐ 14 Other, specify:__________________________________
☐ 777 Don't know/unsure
☐ 999 Refused

For the next questions, I'd like your opinions about Café 55 at Hub 55 on 55th and St. Clair. This can be based on your experience or on what others have said about Café 55.

Please turn to response chart C in your food amounts booklet. Please tell me how you would rate the following questions from poor to excellent.

If participant does not have response card, say the following: Your responses are poor, fair, good, very good, or excellent.

62. How would you rate the quality of foods at Café 55 at Hub 55?

☐ 1 Poor
☐ 2 Fair
☐ 3 Good
☐ 4 Very Good
☐ 5 Excellent

☐ 777 Don't know/unsure

63. How would you rate the prices of Café 55?

☐ 1 Poor
☐ 2 Fair
☐ 3 Good
☐ 4 Very Good
☐ 5 Excellent
☐ 777 Don't know/unsure

Now I will ask you questions about visiting Goldhorn Brewery.

64. Over the past 12 months, how frequently did you visit Goldhorn Brewery at Hub 55 on 55th and St. Clair? [Interview note: Pick one timeframe that best represents their pattern and record number.]

____ 1 times per day	____ 2 times per week	____ 3 times per month	____4 times per year	___5 Never visited

64a. Number of times:____________________________

65. [IF Q60 = 1-4] What are the two best things about the Goldhorn Brewery? [INTERVIEW NOTE: Write "DK" if participant has no response]

1. ____
2. ____

66. [IF Q60 = 1-4] What are the two things that need to be improved at Goldhorn Brewery? [INTERVIEW NOTE: Write "DK" if participant has no response]

1. ____
2. ____

67. [IF Q60 IS 5] Why didn't you visit Goldhorn Brewery during the past 12 months?

☐ 1 Disabled
☐ 2 Schedule conflict
☐ 3 Location of brewery
☐ 4 Time of brewery
☐ 5 Did not know it was open in community
☐ 6 Weather
☐ 7 Health problems
☐ 8 Did not know anyone attending brewery
☐ 9 Not interested
☐ 10 Don't like what it sells
☐ 11 The place is not for me
☐ 12 Too expensive

☐ 13 Did not feel welcome there
☐ 14 I do not drink
☐ 15 Don't need another bar in this neighborhood
☐ 16 Other, specify:________________________________
☐ 777 Don't know/unsure
☐ 999 Refused

68. Now I'd like for you to think about all parts of Hub 55 together. When you think about the St. Clair Farmers' Market, Café 55, and Goldhorn Brewery all together, how much do you agree or disagree with the following statements? Please refer to response chart A in your booklet.

If participant does not have response card, say the following: Your responses are strongly disagree, tend to disagree, tend to agree, or strongly agree.

	Strongly Disagree	Tend to Disagree	Tend to Agree	Strongly Agree	Don't Know
a. There is a good variety of food options at Hub 55.	1	2	3	4	777
b. The people at Hub 55 make you feel welcome.	1	2	3	4	777
c. The workers at Hub 55 are people from your community.	1	2	3	4	777
d. Other customers at Hub 55 are from your community.	1	2	3	4	777
e. It is convenient to shop at Hub 55	1	2	3	4	777
f. Hub 55 is clean.	1	2	3	4	777
g. You can do one-stop shopping at Hub 55.	1	2	3	4	777
h. Hub 55 seems like part of your community	1	2	3	4	777
i. Hub 55 is a gathering place for meeting people from your community.	1	2	3	4	777

---ASK OF ALL PARTICIPANTS --

INTERVIEWER: Thank you for your responses so far. We are more than halfway done with this survey. Now I'm going to ask you a few questions about you and your family. These will help us better understand participants in the study.

69. If female, to your knowledge, are you now pregnant?
☐ 1 Yes

☐ 2 No

Do not read:

☐ 777 Don't know / Not sure
☐ 999 Refused

70. Are you …?

Please read:

☐ 1 Married
☐ 2 Divorced
☐ 3 Widowed
☐ 4 Separated
☐ 5 Never married

Or

☐ 6 A member of an unmarried or partnered couple (i.e., you live with someone but you are not married)

Do not read:

☐ 999 Refused

71. How many people currently live in your household? Please include yourself. [Note: Include people who stay at the house most days of the week.]
_____ Number of people in household (*Confirm they included themselves)

72. How many of these people are adults 18 years or older? _____________

73. How many of these people are children ages 0-17 years? _____________

74. How often in the **past 12 months,** would you say you were worried or stressed about having enough money to pay your rent/mortgage? Would you say you were worried or stressed…

☐ 1 Always
☐ 2 Usually
☐ 3 Sometimes
☐ 4 Rarely
☐ 5 Never

Do not read:

☐ 8 Not applicable
☐ 777 Don't know / Not sure
☐ 999 Refused

75. How often in the **past 12 months,** would you say you were worried or stressed about having enough money to buy nutritious meals? Would you say you were worried or stressed---

☐ 1 Always
☐ 2 Usually
☐ 3 Sometimes
☐ 4 Rarely

- ☐ 5 Never

Do not read:
- ☐ 8 Not applicable
- ☐ 777 Don't know / Not sure
- ☐ 999 Refused

76. I'm going to read you several statements that people have made about their food situation. For these statements, please tell me whether the statement was often true, sometimes true, or never true for (you/your household) in the last 12 months.

INTERVIEWER NOTE: If one person in household, use "I' "my" and "you" in parentheticals, otherwise, use "we" "our" and "your household"

a. The first statement is, "The food that (I/we) bought just didn't last, and (I/we) didn't have money to get more." Was that often, sometimes, or never true for (you/your household) in the last 12 months, since your last interview?

- ☐ 1 Often true
- ☐ 2 Sometimes true
- ☐ 3 Never true
- ☐ 777 Don't Know
- ☐ 999 Refused

b. "(I/we) couldn't afford to eat balanced meals." Was that often, sometimes, or never true for (you/your household) in the last 12 months?

- ☐ 1 Often true
- ☐ 2 Sometimes true
- ☐ 3 Never true
- ☐ 777 Don't Know
- ☐ 999 Refused

c. In the last 12 months, did (you/you or other adults in your household) ever cut the size of your meals or skip meals because there wasn't enough money for food?

- ☐ 1 Yes
- ☐ 2 No (Skip d)
- ☐ 777 Don't Know (Skip d)

d. [IF YES ABOVE, ASK] How often did this happen—almost every month, some months but not every month, or in only 1 or 2 months?

- ☐ 1 Almost every month
- ☐ 2 Some months but not every month
- ☐ 3 Only 1 or 2 months
- ☐ 777 Don't Know

e. In the last 12 months, did you ever eat less than you felt you should because there wasn't enough money for food?

- ☐ 1 Yes
- ☐ 2 No

☐ 777 Don't Know

f. In the last 12 months, were you ever hungry but didn't eat because there wasn't enough money for food?
☐ 1 Yes
☐ 2 No
☐ 777 Don't Know

77. Have there been any major or significant changes in your life or household in the past 12 months? Such as a major illness, death of a loved one, job loss, or birth of a child.
    ☐ 1 Yes
    ☐ 2 No
    ☐ 777 Don't Know
    ☐ 999 Refuse

If yes, please describe:____

The next questions are about your household and family.

78. How often did you move or change addresses over the past 12 months?
    Record number: ____________ (Never = 0)

79. How long have you stayed or lived at your current address? You can answer in amount of days, month, or years… (Please type "X months" OR "X years" OR "X days")

____ days	____ months	____ years	

80. Do you currently own or rent your home?
    ☐ 1 Own
    ☐ 2 Rent
    ☐ 3 Other arrangement

Do not read:
    ☐ 777 Don't know / Not sure
    ☐ 999 Refused

81. What is the highest grade or year of school you completed?

Read only if necessary:
☐ 1 Never attended school or only attended kindergarten
☐ 2 Grades 1 through 8 (Elementary)
☐ 3 Grades 9 through 11 (Some high school)
☐ 4 Grade 12 or GED (High school graduate)
☐ 5 College 1 year to 3 years (Some college or technical school)
☐ 6 College 4 years or more (College graduate)
☐ 7 Post-graduate training but no degree
☐ 8 Post-graduate degree (MS/MA, PhD, JD, MD)

☐ 999 Refused

82. In the **last 12 months**, what was your annual household income from all sources? I'm going to read you a list of ranges, and I'd like for you to say "STOP" when I get to yours.
☐ 1 Less than $10,000
☐ 2 Between $10,001 and $20,000
☐ 3 Between $20,001 and $30,000
☐ 4 Between $30,001 and $40,000
☐ 5 Between $40,001 and $50,000
☐ 6 Between $50,001 and $75,000
☐ 7 $75,000 or higher

Do not read:
☐ 777 Don't know / Not sure
☐ 999 Refused

83. In the **last 12 months**, did you or anyone who lives in your house receive SNAP or Food Stamp benefits?
☐ 1 Yes
☐ 2 No

Do not read:
☐ 777 Don't know / Not sure
☐ 999 Refused

84. In the **last 12 months**, did you or anyone who lives in your house receive WIC benefits?
☐ 1 Yes
☐ 2 No

Do not read:
☐ 777 Don't know / Not sure
☐ 999 Refused

85. In the **last 12 months**, did you or anyone who lives in your house receive other federal financial assistance; for example, TANF, Medicaid, Disability, SSI? (Medicare is NOT considered federal financial assistance.)
☐ 1 Yes
☐ 2 No

Do not read:
☐ 777 Don't know / Not sure
☐ 999 Refused

86. Are you currently…?
Please read:
☐ 1 Employed for wages
☐ 2 Self-employed
☐ 3 Out of work for 1 year or more
☐ 4 Out of work for less than 1 year
☐ 5 A Homemaker
☐ 6 A Student

- ☐ 7 Retired
- ☐ 8 Unable to work

Do not read:
- ☐ 999 Refused

87. How many hours do you work during a typical week? (*Only ask if response to Q86 was "employed for wages" or "self-employed")*

____

INTERVIEWER: The next questions are about health insurance. We will ask you about health insurance obtained through employment or purchased directly as well as government programs like Medicare and Medicaid that provide medical care or help pay medical bills.

88. During 2016, were you EVER covered by health insurance or some other kind of health care plan?
    - ☐ 1 Yes
    - ☐ 2 No [SKIP TO Q92]
    - ☐ 777 Don't Know/ Not Sure [SKIP TO Q92]
    - ☐ 999 Refused [SKIP TO Q92]

89. During 2016, what kind of health insurance or health care coverage did you have? Include those that pay for only one type of service (nursing home care, accidents, or dental care). Exclude private plans that only provide extra cash while hospitalized. If you have more than one kind of health insurance, tell me all plans that you have.
    - ☐ 1 Private Health Insurance (e.g., Medical Mutual, Aetna, United Healthcare, Cigna)
    - ☐ 2 Medicare
    - ☐ 3 Medi-Gap
    - ☐ 4 Medicaid
    - ☐ 5 State Children's Health Insurance Program (SCHIP)
    - ☐ 6 Military Health Care (Tricare/VA/Champ-VA)
    - ☐ 7 Indian Health Service
    - ☐ 8 State sponsored health plan
    - ☐ 9 Other government program
    - ☐ 10 Single service plan (dental, visit, prescriptions)
    - ☐ 11 No coverage of any type
    - ☐ 12 Other, please specify_________________________
    - ☐ 777 Don't know/not sure
    - ☐ 999 Refused

    89a. Other, please specify____

90. In 2016, was there any time when you did NOT have ANY health insurance or coverage?
    - ☐ 1 Yes
    - ☐ 2 No [SKIP TO Q92]
    - ☐ 777 Don't know/not sure

- ☐ 999 Refused

91. During which months in 2016 did you NOT have ANY health insurance or coverage?
    - ☐ January 2016
    - ☐ February 2016
    - ☐ March 2016
    - ☐ April 2016
    - ☐ May 2016
    - ☐ June 2016
    - ☐ July 2016
    - ☐ August 2016
    - ☐ September 2016
    - ☐ October 2016
    - ☐ November 2016
    - ☐ December 2016

92. In the past 12 months, that would be since your last interview, has a doctor, nurse, or other health professional told you that you have any of the following?

a. High blood pressure or hypertension	☐ 1 Yes<br>☐ 2 No
b. Heart disease	☐ 1 Yes<br>☐ 2 No
c. Diabetes/sugar, pre-diabetes, or borderline diabetes	☐ 1 Yes<br>☐ 2 No
d. Obesity or overweight	☐ 1 Yes<br>☐ 2 No
e. Cancer	☐ 1 Yes<br>☐ 2 No
f. Kidney disease	☐ 1 Yes<br>☐ 2 No
g. Asthma	☐ 1 Yes<br>☐ 2 No<br>☐ 777 Don't know
h. Hyperlipidemia/High Cholesterol	☐ 1 Yes<br>☐ 2 No<br>☐ 777 Don't know
i. Other health issue 1: ______________	☐ 1 Yes<br>☐ 2 No
j. Other health issue 2: _______________	☐ 1 Yes<br>☐ 2 No

93. In the past 12 months, that would be since your last interview, has a doctor, nurse, or other health professional told another member of your household that they have any of the following?

If participant says "yes" to any disease, make sure to ask the following question:

94. What is your relationship to this person?

(Skip to Q 95 if no one else lives in household.)

		If yes, what is your relationship to this person?
a. High blood pressure or hypertension	☐ 1 Yes<br>☐ 2 No<br>☐ 777 Don't know	☐ 5 Child<br>☐ 6 Spouse or partner<br>☐ 7 Other family member<br>☐ 8 Roommate<br>☐ 9 Friend<br>☐ 10 Other
b. Heart disease	☐ 1 Yes<br>☐ 2 No<br>☐ 777 Don't know	☐ 5 Child<br>☐ 6 Spouse or partner<br>☐ 7 Other family member<br>☐ 8 Roommate<br>☐ 9 Friend<br>☐ 10 Other
c. Diabetes/sugar, pre-diabetes, or borderline diabetes	☐ 1 Yes<br>☐ 2 No<br>☐ 777 Don't know	☐ 5 Child<br>☐ 6 Spouse or partner<br>☐ 7 Other family member<br>☐ 8 Roommate<br>☐ 9 Friend<br>☐ 10 Other
d. Obesity or overweight	☐ 1 Yes<br>☐ 2 No<br>☐ 777 Don't know	☐ 5 Child<br>☐ 6 Spouse or partner<br>☐ 7 Other family member<br>☐ 8 Roommate<br>☐ 9 Friend<br>☐ 10 Other
e. Cancer	☐ 1 Yes<br>☐ 2 No<br>☐ 777 Don't know	☐ 5 Child<br>☐ 6 Spouse or partner<br>☐ 7 Other family member<br>☐ 8 Roommate<br>☐ 9 Friend<br>☐ 10 Other
f. Kidney disease	☐ 1 Yes<br>☐ 2 No<br>☐ 777 Don't know	☐ 5 Child<br>☐ 6 Spouse or partner<br>☐ 7 Other family member<br>☐ 8 Roommate

		☐ 9 Friend<br>☐ 10 Other
g. Asthma	☐ 1 Yes<br>☐ 2 No<br>☐ 777 Don't know	☐ 5 Child<br>☐ 6 Spouse or partner<br>☐ 7 Other family member<br>☐ 8 Roommate<br>☐ 9 Friend<br>☐ 10 Other
h. Hyperlipidemia/High Cholesterol	☐ 1 Yes<br>☐ 2 No<br>☐ 777 Don't know	☐ 5 Child<br>☐ 6 Spouse or partner<br>☐ 7 Other family member<br>☐ 8 Roommate<br>☐ 9 Friend<br>☐ 10 Other
i. Other health issue 1: ______________	☐ 1 Yes<br>☐ 2 No<br>☐ 777 Don't know	☐ 5 Child<br>☐ 6 Spouse or partner<br>☐ 7 Other family member<br>☐ 8 Roommate<br>☐ 9 Friend<br>☐ 10 Other
j. Other health issue 2: _______________	☐ 1 Yes<br>☐ 2 No<br>☐ 777 Don't know	☐ 5 Child<br>☐ 6 Spouse or partner<br>☐ 7 Other family member<br>☐ 8 Roommate<br>☐ 9 Friend<br>☐ 10 Other

95. Have you smoked at least 100 cigarettes in your entire life? (NOTE: 5 packs = 100 cigarettes)

☐ 1 Yes
☐ 2 No
☐ 777 Don't know / Not sure
☐ 999 Refused

96. [SKIP IF Q 91=2,777, OR 999]] Do you now smoke cigarettes every day, some days, or not at all?

☐ 1 Every day
☐ 2 Some days
☐ 3 Not at all
☐ 777 Don't know / Not sure
☐ 999 Refused

97. About how much do you weigh without shoes in pounds?

___ ____ ____ ____ weight

Do not read:

☐ 777 Don't know / Not sure

☐ 999 Refused

97a. About how tall are you without shoes in feet/inches? (Please enter height as follows “XX/XX”. If an individual is 5’7”, you would enter “05/”07”)

___ ____ ____ ____ height

97b. About how tall are you without shoes in feet/inches? (Please enter height in inches. If an individual is 5’7”, you would enter “67”)

____ ____ height

INTERVIEWER: We are now to the last set of questions. Please think about your neighborhood as the area within a 20 minute walk or 1 mile from your home when answering these.

98. Please turn to response chart A in your food amounts booklet. Please tell me how much you agree or disagree with the following statements when thinking about the last 12 months.

If participant does not have response card, say the following: Your responses are strongly disagree, tend to disagree, tend to agree, or strongly agree.

	Strongly Disagree	Tend to Disagree	Tend to Agree	Strongly Agree	Don’t Know
a. I have pride in the home that I live in.	1	2	3	4	777
b. I have pride in the appearance of the yards in my neighborhood.	1	2	3	4	777
c. I have pride in how the exterior of the homes in my neighborhood look.	1	2	3	4	777
d. I take pride in my neighborhood.	1	2	3	4	777

99. Thinking about your neighborhood overall, please rate how much you agree or disagree with the following statements about your neighborhood? Please refer to Section A of your response chart.

If participant does not have response card, say the following: Your response choices are strongly disagree, tend to disagree, tend to agree, or strongly disagree.

INTERVIEWER: That was our last question for today. Do you have anything else you want to share before we complete the interview survey for today?

____
____
____

	Strongly Disagree	Tend to Disagree	Tend to Agree	Strongly Agree	Don't Know
a. You can get what you need in your neighborhood.	1	2	3	4	777
b. Your neighborhood helps you fulfill your needs.	1	2	3	4	777
c. You feel like a member of your neighborhood.	1	2	3	4	777
d. You belong in your neighborhood.	1	2	3	4	777
e. You have a say about what goes on in your neighborhood.	1	2	3	4	777
f. People in your neighborhood are good at influencing each other.	1	2	3	4	777
g. You feel connected to your neighborhood.	1	2	3	4	777
h. There is a strong sense of community spirit in your neighborhood	1	2	3	4	777
i. You believe your neighborhood is changing for the better.	1	2	3	4	777
j. Your neighborhood seems like the kind of place where one person can make a difference.	1	2	3	4	777
k. Your neighborhood is the kind of place you'd like to live.	1	2	3	4	777

We will mail your gift card within the next 7 business days. We will mail the gift card to the following address [primary address from contact sheet]. Is this okay? Please make sure this is a reliable address, foodNEST is not responsible for any lost or stolen gift cards.

- ☐ Yes
- ☐ No

Okay, what is the correct address?

Please enter Street Address (including apartment/unit number), City, State, and ZipCode. Make sure to double check spelling.

____

The last thing we need to do is review the dates and times of your second and third phone surveys.

Survey 5 (3-10 days from Survey 4)
________________ Date _________________ Day of Week __________ am/pm

Survey 6 (10-30 days from Survey 4)
________________ Date _________________ Day of Week __________ am/pm

Are these two phone numbers still the best to reach you for Survey 5 and 6?
______________________________________ phone 1
______________________________________ phone 2

If you have any questions before then or need to reschedule your next phone survey, feel free to contact the research team at 216-844-1221. As a reminder, you will receive a $35 VISA gift card after completing your second phone survey and $40 VISA gift card after completing your third.

Do you have any questions before we end our conversation today?

Thanks!

Date survey 4 was completed:

SURVEY RESPONSE CARD

	1	2	3	4	5
**Section A**	Strongly disagree	Tend to disagree	Tend to agree	Strongly agree	---
**Section B**	Never	Hardly ever	Now and again	Quite often	Most of the time
**Section C**	Poor	Fair	Good	Very Good	Excellent
**Section D**	Not at all interested	Somewhat interested	Very interested	---	----
